# Modeling CNS Development and Disease

**DOI:** 10.1155/2016/3241057

**Published:** 2016-08-24

**Authors:** Jason P. Weick, Jason S. Meyer, Julia Ladewig, Weixiang Guo, Yan Liu

**Affiliations:** ^1^Department of Neurosciences, University of New Mexico-HSC, Albuquerque, NM 87131-0001, USA; ^2^Department of Biology/Stark Neurosciences Research Institute, Indiana University-Purdue University Indianapolis, Indianapolis, IN 46202, USA; ^3^Institute of Reconstructive Neurobiology, University of Bonn, 53127 Bonn, Germany; ^4^Institute of Genetics and Developmental Biology, Chinese Academy of Sciences, Beijing, China; ^5^Institute for Stem Cell and Neural Regeneration, School of Pharmacy, Nanjing Medical University, Nanjing, China

The ultimate goals for stem cell researchers are uncovering the fundamental mechanisms of cellular biology as well as developing treatments for disease and/or injury. The central nervous system (CNS) presents an unparalleled challenge for these studies due to the diversity of cell types and their interrelated functions in health and disease. Furthermore, the endpoints for determining phenotypic maturation are complicated by the fact that cells, especially neurons, express a plethora of receptors and ion channels that control excitability and plasticity which vary greatly between CNS regions. For this reason, extensive effort has been placed on developing methods to differentiate appropriate populations of cells that reside within the CNS (e.g., neuronal subtypes, astrocytes, and oligodendrocytes). These studies typically employ phenotypic validation by the use of expression analysis for various protein markers of regionalized populations, with a burgeoning effort to verify functional outcomes. Much of this work has been informed by basic developmental studies that have identified cell-intrinsic and cell-extrinsic signaling mechanisms, as well as transcriptional programs involved with cell fate specification.

While early work focused on the use of mesenchymal stem cells, embryonic carcinoma cells, and fetal stem cells, more recent efforts have focused on the areas of human embryonic stem cells (hESCs) and induced pluripotent stem cells (iPSCs). These sources typically require slightly different methods to direct differentiation to various CNS fates, although many mechanisms appear to converge on common signaling pathways. Many recent studies have even successfully uncovered cell-type specific phenotypes that are appropriate for various human diseases. However, despite these advances, limitations still hamper the application of human stem cells for CNS development/disease studies. First, most differentiation protocols lead to a heterogeneity of cells in terms of both their “regional” markers and their maturational states. Furthermore, the number and diversity of available fate-specific biomarkers remain insufficient to unambiguously identify regional and transmitter phenotypes.

In this special issue, we highlight both the advancements and challenges of directed differentiation of various neuronal and glial cells from pluripotent stem cells. We hope this collection of works will provide readers with a resource to understand directed differentiation, functional maturation, and transplantation-induced changes to host environments.

The article by P. Prajumwongs et al. reviews the current state of knowledge regarding neural induction of human pluripotent stem cells, detailing the signaling pathways and timing of critical events such as neuroepithelial differentiation. Emphasis is placed on the comparison between* in vitro* and* in vivo* development and the utility of hESCs to model human-specific signaling processes. The article concludes with a series of brief descriptions about various developmental and neurodegenerative diseases and how iPSCs have been used to model their underlying molecular and cellular pathologies.

The review article from A. Zirra et al. gives a detailed overview of the development of the neural tube and how these* in vivo* principles provided the basis for* in vitro* neural induction strategies of human pluripotent stem cells (hPSCs). The article highlights the basic stages of neural development including neural induction, neurulation, and neural patterning. Here they focus on signaling pathways, morphogenes, and patterning factors described to be crucial for the development of distinct brain regions. The authors then discuss how knowledge from* in vivo* brain development has been translated by many groups to direct the differentiation of hPSCs to clinically relevant region-specific neurons* in vitro*. Finally, they give an outlook of how human brain development data can be utilized for improving directed differentiation protocols and validating the thereof derived region-specific subpopulations of human neurons.

The article by F. Cavaliere et al. reviews the use of organotypic hippocampal slice cultures as a model for understanding neurogenesis under normal and pathological conditions. They first present a historical summary of the development of slice cultures from neonatal, and more recently adult animals, for the study of neurogenesis from both SVZ and DGZ. The article also describes results from pathological studies of slice cultures in the context of ischemia, Parkinson's, and Alzhemier's disease, as well as regeneration of neurons in the spinal cord. The authors suggest that the maintenance of the 3D architecture and cellular composition of these cultures make them unique among* in vitro* models for these types of studies.

The primary research article from T. H. Shin et al. tested the effects of mesenchymal stem cells on polyamine levels in both* in vitro* and* in vivo* models of ischemic stroke. Polyamines are low molecular weight compounds secreted in high concentrations from the brain and are important indicators of metabolic function, especially in response to injury or disease. The authors found that middle cerebral artery occlusion (MCAo)* in vivo* and oxygen-glucose deprivation (OGD) of a neural cell line* in vitro* significantly altered a plurality of polyamine levels. Importantly, the authors found that disruptions of polyamine levels by MCAo- and OGD-induced ischemic conditions could be restored to near-normal levels after introduction of human bone marrow-derived mesenchymal stem cells.

Finally, the review article from J. P. Weick highlights the efforts by many groups to understand the functional phenotypes of pluripotent stem cell-derived neurons as well as directly converted/induced neurons (iNs). This article focuses on the assessment of developmental maturation of forebrain neurons via analysis of forebrain glutamatergic and GABAergic currents, comparing these* in vitro* findings to* in vivo* data from rodent models. The author then discusses recent results modeling various neurological disorders using iPSC-derived neurons and iNs. He concludes by critically reflecting on the use of these two cell types for developmental and disease studies and provides suggestions on how to optimally determine and report neuronal function.

Together, the articles in this special issue highlight recent advances in our understanding of the directed differentiation and functional properties of stem cells and describe several new approaches to model and develop novel therapies for neurological disease and injury.

*Jason P. Weick* *Jason P. Weick* *Jason S. Meyer* *Jason S. Meyer* *Julia Ladewig* *Julia Ladewig* *Weixiang Guo* *Weixiang Guo* *Yan Liu* *Yan Liu*

